# Brief communication: The rate of switching from first-line to second-line antiretroviral therapy among people living with HIV in Aden City, Yemen

**DOI:** 10.1186/s12981-024-00638-z

**Published:** 2024-07-30

**Authors:** Naif Mohammed Al-Haidary, Enas Abobakr Radman

**Affiliations:** 1 Department of Medical Microbiology and Immunology, Faculty of Medicine and Health Sciences, Sana’a University, Sana’a, Yemen; 2 International Rescue Committee, Aden, Yemen

**Keywords:** HIV, Antiretroviral therapy, Second-line treatment, ART switching, Yemen

## Abstract

**Background:**

Effective management of antiretroviral therapy (ART) is crucial in combating the global HIV pandemic. This study, the first of its kind in Yemen, investigates the rate and determinants of switching from first-line to second-line ART among people living with HIV (PLWH) in Aden City, Yemen.

**Methods:**

A retrospective cohort study was conducted using data from PLWH who started first-line ART at Al-Wahda Hospital from 2007 to May 2022. PLWH in prevention of mother-to-child transmission (PMTCT) programs, those already on second-line ART at enrollment, and those with less than 3 months of follow-up were excluded. Cumulative incidence curves and multivariable proportional hazards models were used to identify factors associated with switching, considering death and loss to follow-up as competing risks. Analyses were carried out using IBM SPSS version 26.

**Results:**

Out of 149 PLWH, 18 (12.1%) switched to second-line ART with a cumulative incidence rate of 1.8 per 100 person-years. Significant factors for switching included being older than 33 years (HR: 1.45, 95% CI: 1.12–1.89), having WHO stage 3 disease (HR: 1.58, 95% CI: 1.21–2.06), and being on a TDF-FTC-EFV-based first-line regimen (HR: 1.35, 95% CI: 1.03–1.77). This switching rate is consistent with rates observed in other resource-limited settings, indicating it is neither exceptionally high nor low compared to similar contexts​.

**Conclusions:**

The study highlights key factors associated with switching to second-line ART in Yemen, emphasizing the need for targeted interventions and continuous monitoring to enhance treatment outcomes. These findings are consistent with regional data from other resource-limited settings.

## Introduction

HIV has been a significant global health challenge since its identification in the 1980s, leading to high morbidity and mortality [1–3]. Antiretroviral therapy (ART) has revolutionized HIV treatment, significantly improving the lifespan and quality of life for those infected [1, 2]. However, the durability of first-line ART regimens and the need for second-line treatments remain critical, especially in resource-limited settings like Yemen [2, 4].

In Yemen, managing HIV is particularly challenging due to limited healthcare resources, ongoing conflicts, and fragile infrastructure. The country has been in conflict since 2015, which has severely impacted the healthcare system. In 2007, HIV treatment services were established in only five out of twenty-two main governorates, resulting in low access to services and poor treatment outcomes [5]. These five centers, originally interconnected, are now under the control of different groups involved in the conflict. This separation has further disrupted the healthcare infrastructure, not only destroying health systems but also isolating these centers from one another. The city’s only facility providing ART free of charge for PLWH is located in Al-Wahda Hospital, operating under the National AIDS & STIs Control Program in cooperation with the WHO [6]. Despite these efforts, little is known about the situation of HIV infection in Yemen. Previous studies have shown that ART services are limited and often disrupted due to internal conflicts, affecting treatment outcomes and leading to higher rates of switching to second-line regimens [5, 7].

There is a significant gap in knowledge regarding the specific factors that contribute to ART switching in Yemen. While regional studies provide some insights, no comprehensive studies have been conducted in Yemen to systematically investigate the determinants of switching from first-line to second-line ART. Understanding these factors is crucial for optimizing treatment strategies and improving patient outcomes in this resource-limited setting.

This study, the first of its kind in Yemen, aims to assess the rate and determinants of switching from first-line to second-line ART among PLWH in Aden City. Understanding these factors is essential for optimizing treatment strategies, improving PLWH outcomes, and supporting the national HIV program in Yemen. By integrating findings from similar studies in resource-limited settings, this research provides a comprehensive view of the challenges and potential solutions for HIV treatment in Yemen [5, 7].

## Methods

### *Study Design and Population*

This retrospective cohort study included PLWH who began first-line ART at Al-Wahda Hospital from 2007 to May 2022. PLWH in PMTCT programs, those already on second-line ART at enrollment, and those with less than 3 months of follow-up were excluded. De-identified data were extracted from the National AIDS & STIs Control Program database, which includes comprehensive clinical and demographic information.

### *Data Collection and statistical analysis*

Data on PLWH demographics, clinical characteristics, and ART regimens were gathered. Switching was determined based on clinical criteria. Due to the ongoing conflict in our country, routine measurements of CD4 count and viral load are not feasible. Descriptive statistics were utilized to illustrate the frequency, percentage, mean, range, median, interquartile range (IQR) and standard deviation (SD). The distribution of continuous variables was explored to guide their categorization, and chi-square tests were used for comparing categorical variables when the expected frequencies were sufficient. For categorical variables with small sample sizes or expected frequencies less than 5, Fisher’s exact test was used.

Participants started accumulating person-time from the initiation of antiretroviral therapy (ART). Cumulative incidence curves were utilized to estimate the time to switching, while multivariable proportional hazards models were employed to identify factors associated with switching, taking into account death and loss to follow-up as competing risks. To identify factors associated with switching to the second-line ART, we initially conducted univariate binary logistic regression, selecting independent variables with a p-value of less than 0.25 for inclusion in the multiple logistic regression analysis. In the multiple logistic regression analysis, an association between the outcome and the independent variables was considered significant at *P* < 0.05. The final model showed good predictive ability, with the likelihood ratio test, Wald test, and score (log rank) test all demonstrating the significance of the model. Statistical analyses were conducted using IBM SPSS Statistics for Windows, version 26.0 (IBM Corp., Armonk, NY, USA). Cases with missing data were minimal and excluded from the analysis to maintain the accuracy and robustness of the results.

### Ethical considerations

#### Ethical approval

was obtained from the Al-Wahda Hospital Ethics Committee and the National AIDS & STIs Control Program. PLWH confidentiality was maintained by anonymizing the data.

This study utilized anonymized data extracted from the National AIDS & STIs Control Program database. While the data was de-identified to maintain patient confidentiality, individual patient consent was not obtained due to the retrospective nature of the study and the specific context in Yemen. Conducting studies involving PLWH in Yemen is particularly challenging due to the sensitivity of the patients and their fear of the social stigma associated with HIV infection. This stigma significantly impacts the willingness of individuals to participate in research and disclose their health status. The authorities and ART program employees in Yemen prioritize protecting patient identities to prevent further stigmatization and social repercussions. Consequently, all information about the disease, its patients, and their treatment is carefully guarded. While this approach helps protect patient privacy and confidentiality, it also raises ethical questions about patient autonomy and the right to be informed about how personal health information is used. In this context, the use of anonymized data is a pragmatic solution that complies with ethical standards for research in resource-limited settings. Nevertheless, future studies should aim to balance the need for comprehensive data collection with the ethical requirement of obtaining informed consent whenever feasible, and efforts should be made to educate the community to reduce stigma and improve participation in research.

## Results

### Socio-demographic characteristics

The study included 149 PLWH, with 63.8% being male and a mean age of 33 years. A third of the PLWH were women (36.2%), and notably, almost half of them (44.4%) were pregnant. The socio-demographic and clinical characteristics, as well as the risk factors associated with the likelihood of switching to second-line ART, are detailed in Table 1.

**Table 1:** Baseline characteristics of people living with HIV (PLWH) and factors associated with switching to second-line ART in Aden City, Yemen, 2007–2022

Characteristics	Switched (*N* = 18)	%	Did Not Switch (*N* = 131)	%	*p*-value
**Gender**					0.78
Male	11	61.1%	84	64.1%	
Female	7	38.9%	47	35.9%	
**Age**					0.12
< 10	0	0.0%	11	8.4%	
10–19	0	0.0%	8	6.1%	
> 19	18	100.0%	112	85.5%	
**Marital status**					0.32
Single	4	22.2%	44	33.6%	
Married	13	72.2%	84	64.1%	
Widowed	0	0.0%	1	0.8%	
Divorced/Separated	1	5.6%	1	0.8%	
Missing data	0	0.0%	1	0.8%	
**Education**					0.64
None	5	27.8%	17	13.0%	
Less than primary	1	5.6%	10	7.6%	
Primary	7	38.9%	55	42.0%	
Secondary	5	27.8%	45	34.4%	
More than secondary	0	0.0%	4	3.1%	
**Employment**					0.80
Not employed	12	66.7%	83	63.4%	
Government sector	0	0.0%	8	6.1%	
Private sector	6	33.3%	40	30.5%	
**WHO stage**					**0.003**
Stage 1	3	16.7%	44	33.6%	
Stage 2	0	0.0%	9	6.9%	
Stage 3	11	61.1%	49	37.4%	
Stage 4	4	22.2%	27	20.6%	
Missing data	0	0.0%	2	1.5%	
**Pregnancy**					**0.033**
Pregnant	0	0.0%	24	51.1%	
Not pregnant	7	100.0%	23	48.9%	
**ART regimen**					**0.004**
AZT-3TC-EFV	4	22.2%	26	19.8%	
AZT-3TC-NVP	1	5.6%	21	16.0%	
D4T-3TC-EFV	0	0.0%	5	3.8%	
TDF-FTC-DTG	0	0.0%	22	16.8%	
TDF-FTC-EFV	12	66.7%	47	35.9%	
Other regimens	1	5.6%	10	7.6%	
**Duration on first-line ART**					0.105
< 45 months	7	38.9%	36	27.5%	
45–94 months	8	44.4%	39	29.8%	
> 94 months	3	16.7%	56	42.7%	
**ART regimen change**					0.08
No	2	11.1%	36	27.5%	
Once	10	55.6%	59	45.0%	
More than once	6	33.3%	36	27.5%	
**Co-infections**					0.72
Hepatitis B Virus	0	0.0%	2	1.5%	
Hepatitis C Virus	0	0.0%	1	0.8%	
Tuberculosis	1	5.6%	8	6.1%	
**Factors associated with switching to second-line ART**					
**Socio-demographic** **factors**		**Hazard Ratio** **(95% CI)**	**p-value**	**Adjusted** **Hazard Ratio** **(95% CI)**	**p-value**
Age groups	=< 33 years	ref.			
	> 33 years	3.6 (1.2–10.9)	0.024	3.9 (1.1–13.5)	**0.029**
Literacy	Illiterate	ref.			
	literate	0.4 (0.15–1.2)	0.11	0.3 (0.1-1.0)	0.056
**Baseline clinical factors**					
WHO stage	Stage 3	3.3 (1.2–9.2)	0.025	4.7 (1.5–14.1)	**0.006**
	Other stages	ref.			
ART regimen	TDF-FTC-EFV	4.1 (1.4–12.1)	0.01	7.6 (2.1–27.0)	**0.002**
	Other regimens	ref.			

ART: Antiretroviral Therapy; AZT: Zidovudine; 3TC: Lamivudine; EFV: Efavirenz; NVP: Nevirapine; D4T: Stavudine; TDF: Tenofovir Disoproxil Fumarate; FTC: Emtricitabine; DTG: Dolutegravir; WHO: World Health Organization; CI: Confidence Interval

p-values in bold indicate statistical significance

### Incidence of switching

**Fig. 1:**
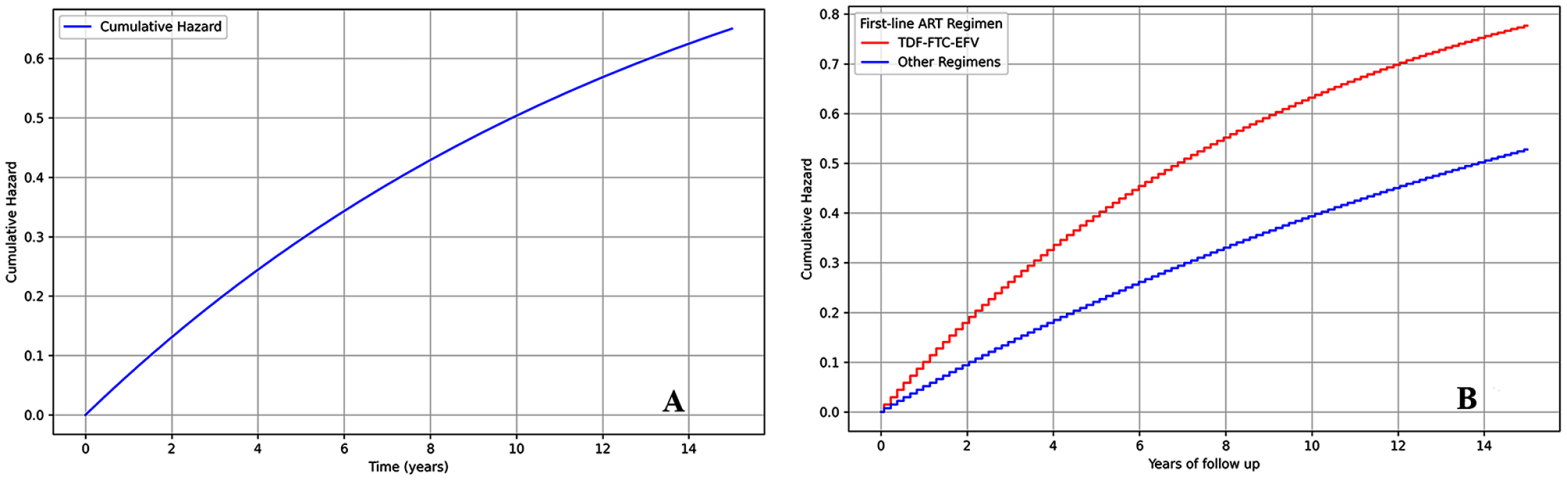
Cumulative Hazard of Switching to Second-line ART Among PLWH in Aden City, Yemen, 2007–2022. (**A**): Overall cumulative hazard of switching to second-line ART. (**B**): Cumulative hazard of switching to second-line ART by first-line ART regimen (TDF-FTC-EFV vs. other regimens). *Abbreviations*: PLWH, People Living with HIV; ART, Antiretroviral Therapy; TDF, Tenofovir Disoproxil Fumarate; FTC, Emtricitabine; EFV, Efavirenz

Out of the 149 PLWH who started with first-line ART, 18 (12.1%) switched to second-line ART. They were followed up for a total of 995 person-years, resulting in an incidence rate of switching to second-line ART of 1.8 per 100 person-years of retrospective follow-up (PYFU). The mean survival time for the entire cohort was 13.57 years (95% CI: 12.7–14.4 years) with a median follow-up time of 5.0 years and an interquartile range (IQR) of 3.2 years. The cumulative hazard of switching to second-line ART increased with the duration on first-line ART, reaching nearly 5% by the end of the third year, as shown in Fig. 1A.

### Factors associated with switching to second-line ART

Statistically significant factors included age over 33 years, WHO stage 3 disease, and being on a TDF-FTC-EFV-based first-line regimen. The hazard ratios and confidence intervals are listed in Table 1.

Compared to those who were 33 years or younger, those older than 33 years had a higher risk of switching to second-line ART. The adjusted hazard ratio for this age group was 3.9 (95% CI: 1.1–13.5, *p* = 0.029), indicating a significantly higher risk of switching among older patients.

Patients with WHO stage 3 disease had a significantly higher risk of switching compared to those in other stages. The adjusted hazard ratio for WHO stage 3 was 4.7 (95% CI: 1.5–14.1, *p* = 0.006), underscoring the increased likelihood of switching in more advanced disease stages.

Those on a TDF-FTC-EFV-based first-line regimen had a higher risk of switching compared to those on other regimens. The adjusted hazard ratio for the TDF-FTC-EFV regimen was 7.6 (95% CI: 2.1–27.0, *p* = 0.002), indicating a significantly higher risk of switching associated with this regimen. The cumulative hazard of switching to second-line ART by first-line ART regimen, comparing TDF-FTC-EFV to other regimens, is illustrated in Fig. 1B.

## Discussion

This study provides important insights into the rate and determinants of switching from first-line to second-line ART among PLWH in Aden City, Yemen. It is the first of its kind in Yemen, paving the way for wider studies and providing valuable insights into the challenges faced by PLWH. The overall incidence of switching was found to be 1.8 per 100 person-years of follow-up. Significant factors for switching included age over 33 years, WHO stage 3 disease, and a TDF-FTC-EFV-based first-line regimen.

The overall incidence of switching was found to be 1.8 per 100 person-years of follow-up, aligning with other studies from resource-limited settings where ART monitoring is less rigorous and access to second-line therapies can be limited [9, 10]. Comparative data from similar settings show comparable switching rates, highlighting the need for enhanced monitoring in resource-limited settings [8]. For example, in a cohort study conducted in Yemen, the rate of treatment failure and switching was similarly high, underscoring systemic challenges in maintaining effective ART regimens [5].

Age over 33 years was a significant factor for switching, consistent with other studies where advanced age is linked to higher rates of treatment failure and switching due to increased comorbidities and drug resistance [5, 7].

WHO stage 3 disease was another significant factor for switching. This stage is associated with more advanced disease and a higher likelihood of treatment failure, necessitating a switch to second-line therapy. This finding is in line with previous research that associates advanced disease stages with increased switching rates [5, 7].

The association between the TDF-FTC-EFV regimen and switching may be attributed to the higher propensity for resistance development with this combination, as documented in several studies [11, 12]. This underscores the need for robust treatment monitoring systems to identify and address treatment failures early, as recommended by WHO guidelines [13].

The findings underscore the importance of targeted interventions and continuous monitoring to enhance treatment outcomes [14]. Implementing routine viral load monitoring, despite financial and logistical constraints, could lead to timely interventions and improve PLWH outcomes in Yemen [5–7].

Tailored interventions for older PLWH and those with advanced disease stages are crucial. Enhancing public education and reducing stigma through community engagement can support better health-seeking behaviors and adherence to ART [7, 13].

This study’s strengths include the use of a well-defined cohort and comprehensive data collection, which enhance the reliability of the findings. However, the limitations include its retrospective nature and reliance on medical records, which may not capture all relevant variables. Unfortunately, we did not have sufficient data on adherence to include it in our analysis. This limitation is due to the retrospective nature of our study, which relied on pre-existing records that did not consistently document adherence information. We acknowledge that adherence could be a significant factor influencing switching and a potential confounder for other associated factors.

Ideally, people who are switched tend to be sicker than those who are not switched. This tends to introduce what is called confounding by indication, which requires rigorous analysis beyond what has been done [15]. In our analysis, we used binary logistic regression and Cox proportional hazards models to identify predictors of switching from first-line to second-line ART. We recognize that this approach may not fully account for confounding by indication, where sicker patients are more likely to be switched to second-line therapy. We attempted to apply Cox proportional hazard marginal structural models (MSMs) but encountered several challenges due to the small sample size and low event count in our dataset. Specifically, the application of MSMs was not feasible given these constraints. While the current methods provided significant insights, we acknowledge the importance of MSMs and plan to incorporate such advanced methods in future research, given the appropriate data conditions.

## Conclusion

This study underscores the importance of continuous monitoring, targeted interventions, and public education in managing HIV in resource-limited settings like Yemen. The key findings highlight that age over 33 years, WHO stage 3 disease, and TDF-FTC-EFV-based regimens are significant predictors of switching to second-line ART. By addressing these specific factors, healthcare providers can improve ART outcomes and enhance the quality of life for PLWH in Yemen.

## Recommendations

1. Conduct Larger Studies: Larger cohort studies across multiple sites are needed to confirm these findings and identify additional factors influencing ART switching.
2. Ensure Equal Access to ART: Implement policies to ensure equal access to ART for all demographics, including marginalized groups.
3. Empower PMTCT Programs: Strengthen PMTCT programs to prevent mother-to-child transmission of HIV.
4. Enhance Public Education: Increase public education and awareness about HIV and the importance of ART adherence.
5. Implement Family Support Programs: Develop family support programs to assist PLWH in maintaining adherence to ART.

Future research should build on these findings to provide a comprehensive understanding of treatment dynamics in diverse settings, ultimately supporting the national HIV program in Yemen.

## Data Availability

The datasets used and/or analyzed during the current study are available from the corresponding author on reasonable request.

## Abbreviations

3TC: Lamivudine
ART: Antiretroviral Therapy
AZT: Zidovudine
CD4: Cluster of Differentiation 4
CI: Confidence Interval
D4T: Stavudine
DTG: Dolutegravir
EFV: Efavirenz
FTC: Emtricitabine
HR: Hazard Ratio
IQR: Interquartile Range
MSMs: Marginal Structural Models
NVP: Nevirapine
PLWH: People Living with HIV
PMTCT: Prevention of Mother-to-Child Transmission
PYFU: Person-Years of Follow-Up
SD: Standard Deviation
SPSS: Statistical Package for the Social Sciences
STIs: Sexually Transmitted Infections
TDF: Tenofovir Disoproxil Fumarate
WHO: World Health Organization
